# Optimization and workflow of *in vitro* culture of adult *Fasciola hepatica*

**DOI:** 10.1590/S1984-29612024064

**Published:** 2024-11-22

**Authors:** Cesar Burga-Cisterna, Edith Málaga, Enrique Serrano-Martínez, Giovana Livia-Córdova, Ramón Antezana, Américo Castro Luna, Monica Pajuelo

**Affiliations:** 1 Laboratorio de Parasitología Animal, Facultad de Medicina Veterinaria y Zootecnia, Universidad Peruana Cayetano Heredia, Lima, Perú; 2 Laboratorios de Investigación y Desarrollo, Facultad de Ciencias e Ingeniería, Universidad Peruana Cayetano Heredia, Lima, Perú; 3 Laboratorio de Parasitología, Facultad de Medicina Veterinaria, Universidad Nacional Pedro Ruiz Gallo, Lambayeque, Perú; 4 Departamento de Veterinaria, Frigorífico Camal San Pedro, Lima, Perú; 5 Instituto de Investigación en Ciencias Farmacéuticas y Recursos Naturales, Facultad de Farmacia y Bioquímica, Universidad Nacional Mayor de San Maros, Lima, Perú; 6 Departamento de Ciencias Celulares y Moleculares, Facultad de Ciencias e Ingeniería, Universidad Peruana Cayetano Heredia, Lima, Perú

**Keywords:** In vitro, Fasciola hepatica, culture media, DMSO, In vitro, Fasciola hepatica, meio de cultura, DMSO

## Abstract

The aim of this study was to evaluate different transportation and incubation conditions to test the viability of adult *Fasciola hepatica* in order to propose a new cultivation workflow. The adult stage of *F. hepatica* was obtained from naturally infected cattle at a local slaughterhouse in Lima, Peru. Different transport and incubation conditions of *F. hepatica* were tested, evaluating its viability through a motility scale. DMEM and RPMI 1640 media presented better transport conditions compared to Hedon-Fleig and PBS media (*p* < 0.001), maintaining the flukes at 37°C. Also, DMEM and RPMI-1640 media presented better incubation conditions compared to Hedon-fleig (*p* < 0.001). A minimum of 3 ml of medium per fluke was required to maintain best viability (*p* < 0.001) and no differences in viability were found between the different types of culture plates (*p* > 0.05). In addition, we found that incubation with DMSO (dimethyl sulfoxide) at concentrations greater than 0.5% v/v for 48 hours generates toxicity (*p* < 0.001). In conclusion, RPMI 1640 and DMEN media presented better transport and *in vitro* cultivation conditions for *F. hepatica*, using DMSO at concentrations lower than 0.5% v/v.

## Resumo

O objetivo deste estudo foi avaliar diferentes condições de transporte e incubação, para testar a viabilidade de *Fasciola hepatica* adulta, a fim de propor um novo fluxo de cultivo. O estágio adulto de *F. hepatica* foi obtido de bovinos, naturalmente infectados, em um matadouro local em Lima, Peru. Foram testadas várias condições de transporte e incubação de *F. hepatica*, avaliando sua viabilidade por meio de uma escala de motilidade. Os meios DMEM e RPMI 1640 apresentaram melhores condições de transporte em comparação com os meios Hedon-Fleig e PBS (*p* < 0,001), mantendo os vermes a 37°C. Além disso, os meios DMEM e RPMI-1640 apresentaram melhores condições de incubação em comparação ao Hedon-fleig (*p* < 0,001). Foi necessário um mínimo de 3 ml de meio por verme para manter a melhor viabilidade (p < 0,001) e não foram encontradas diferenças na viabilidade entre os diferentes tipos de placas de cultura (*p* > 0,05). Além disso, foi constatado que a incubação com DMSO (dimetilsulfóxido) em concentrações superiores a 0,5% v/v, por 48 horas, gera toxicidade (*p* < 0,001). Conclui-se que os meios RPMI 1640 e DMEN apresentaram melhores condições de transporte e cultivo *in vitro* para *F. hepatica*, utilizando DMSO em concentrações inferiores a 0,5% v/v.

## Introduction

Fasciolosis is a cosmopolitan parasitic disease that affects human and animal health. It is caused by *Fasciola hepatica*, a trematode whose intermediate hosts are snails of the family Lymnaeidae and definitive hosts are the herbivorous mammals and humans (Livia-Córdova et al., 2021; Alsulami et al., 2023; Juhasz et al., 2023; Rosas-Hostos Infantes et al., 2023). This parasite causes hepatic injuries and results in significant economic losses in the global livestock sector. These losses are estimated to be 3.2 billion dollars per year due to liver confiscation, reduced productivity, and veterinary (Rashid et al., 2019).

Furthermore, the zoonosis risk of fasciolosis is high. It has been estimated that fasciolosis affects approximately 2.4 million people worldwide, mainly in rural areas of the Andean highlands (Caravedo & Cabada, 2020). In parasitized humans, it causes abdominal pain, jaundice and fever (Pfeifer et al., 2019), anemia and cirrhosis (Lopez et al., 2012; Machicado et al., 2016).

Currently, fasciolosis control faces different challenges such as drug resistance (Romero et al., 2019; Morales et al., 2021; Larroza et al., 2023), difficulties in diagnosis (Aftab et al., 2024) and emerging cases that call for investigation. Therefore, *in vitro* studies are essential for the research on diagnosis and treatment. Parasite cultivation techniques constitute basic elements for first steps research in those areas. Currently, there is no specific culture media for *F.hepatica*, but the use of different cell culture media such as Hedon-Fleigh (Singh et al., 2009), RPMI 1640 (Duthaler et al., 2010; Jeyathilakan et al., 2012; Ullah et al., 2017; Machicado et al., 2019) and M-199 (Tansatit et al., 2012), has been reported. However, the supplements used vary in each study and details of the culture process (transport, sterilization, management flukes, etc.) for successful cultivation, have not been reported in the literature. In addition, in the *F. hepatica* drug testing assay, DMSO in one of the most used solvents; however, it can be toxic (Yi et al., 2017) and it is necessary to know its effect on *F. hepatica* cultivation. Therefore, the aim of this study is to evaluate the viability of *F. hepatica* under different transport and incubation conditions to propose a specific workflow for the culture of *F. hepatica.*

## Material and Methods

Due to the lack of detail in the literature, all transport and cultivation conditions were proposed by this research group, except for the culture media, temperature and microaerophilic conditions, that have already been published (Tansatit et al., 2012; Ullah et al., 2017; Machicado et al., 2019).

### Parasite collection

Adult *F. hepatica* (Valero et al., 2012) were collected directly from the bile ducts of naturally infected cattle that were slaughtered in the slaughterhouse “Frigorífico Camal San Pedro” in Lima, Peru. Groups of fifteen adult flukes were collected from 37 different cattle. *Fasciola gigantica* is not reported in Peru, therefore only *F. hepatica* was collected. Samples were collected by non-probabilistic convenience sampling, collecting the flukes according to the order of arrival of parasitized animals. The sampling was carried out from June to August 2021.

### Transport

Various conditions were tested for the transport of *F. hepatica* from the slaughterhouse to the laboratory (average travel time of 3 hours). DMEM (Dulbecco′s Modified Eagle′s Medium – Sigma Aldrich), Hedon-Fleig (18), RPMI 1640 (Sigma – Aldrich, USA) and PBS were evaluated as transport media. All the media were supplemented with penicillin at 1000 IU/ml and gentamicin at 0.1 mg/ml under sterile conditions. Various thermal conditions were evaluated for their transport: 18 ± 2 °C (environmental temperature according to the Peruvian national weather authority - SENAMHI), 37 ± 2 °C and temperatures higher than 39 °C (until 50°C). 15 adult flukes were placed and transported for each sterile 50ml Falcon tubes containing 40ml of the transport media. The falcon tubes were stored in thermal boxes. All adult *F. hepatica* were transported to the laboratory immediately after their collection from the bile ducts. Viability was evaluated immediately upon arrival at the laboratory, during the washing process before cultivation. Each transport condition was evaluated in duplicate.

### Washing

The washing and cultivation processes were performed in the Laboratory of Research of Infectious Diseases at Universidad Peruana Cayetano Heredia. All collected flukes were washed before cultivation. This process was carried out twice with Hedon-Fleig media supplemented with antibiotics (penicillin at 1000 IU/ml and gentamicin at 0.1 mg/ml) and a last wash was carried out with the same media to be incubated for adaptation. The washing and culture media were maintained at 37°C during the entire process.

### Cultivation

For the cultivation of *F. hepatica*, three types of culture media were evaluated: DMEM, Hedon-Fleig and RPMI-1640. Initially 9 ml of culture media per fluke were incubated in petri dishes for up to 7 days to determinate the maximum *in vitro* survival days. Then, volumes of culture media per fluke (2, 2.5, 3, 4 and 5 ml per well) were evaluated using the best culture media selected in the previous step. Also, different types of culture plates were compared, evaluating 6-well culture plates (with a cell growth area of 9.5 cm^2^ and a maximum volume of 17 ml per well) and 24-well culture plates (with a cell growth area of 1.9 cm^2^ and a maximum volume of 3.5 ml per well). The evaluations of types of culture were conducted using the optimal culture media volume previously selected. Additionally, the influence of DMSO on the viability of *F. hepatica* was evaluated by analyzing concentrations of 0.2, 0.5, 1, 5 and 10% (v/v) in the RPMI-1640 media. Each cultivation condition was evaluated in duplicates.

All cultivations were performed at 37°C, with 5% CO_2_ and 95% humidity (Duthaler et al., 2010; Ullah et al., 2017; Machicado et al., 2019). All the media were kept in sterile conditions, with a pH of 7.4, and were supplemented with penicillin at 1000 IU/ml and gentamicin at 0.1 mg/ml. The entire process of washing and preparation of the *F. hepatica* culture was carried out in laminar flow cabinets under sterile conditions.

### Viability evaluation

The viability of *F. hepatica* was evaluated by the research team and a motility score with the following scale was used:

3 – Good motility (fast movements with good intensity)
2 – Reduced motility (slow movements with low intensity, reduced by 50%).
1 – Very reduced motility (moves only parts of the body, generally in peripheral areas).
0 – Immobile and dead (shows total paralysis and pale coloration, confirmed with a 10X stereoscope).

This motility scale was applied after the washing and incubation process.

### Statistical analyzes

Descriptive statistical of motility score was presented by median, interquartile range, minimum and maximum values. Differences of motility score between different cultivation conditions was evaluated by using the Kruskal-Wallis test and Dunn´s post-hoc test. *P* < 0.05 was considered as statistically significant. All statistical analysis was performed with the Stata software (StataCorp LP, College Station, TX).

## Results

### Transport

The RPMI, DMEM, Hedon-Fleig and PBS media maintained good viability of adult flukes during transport while the temperature of the culture media was approximately of 37°C, showing best viability in RPMI and DMEN media (*p* < 0.001). However, at temperatures higher than 39°C and at environmental temperature, the viability was low, showing better viability RPMI, DMEN and Hedon-Fleig media (motility score = 1) compared with PBS (motility score = 0; *p* < 0.001; Table 1).

**Table 1:** Assessment of the viability in the transport of the adult stage of *Fasciola hepatica* under different conditions.

Temperature	Culture media^*^				
	RPMI 1640 (n=90)	DMEN (n=90)	Hedon-Fleig (n=90)	PBS (n=90)	*p-value* ^**^
Environmental temperature (18°C)	1 (0)	1 (0)	1 (0)	1 (0)	0.232
37°C	3 (0)^ab^	3 (0)^cd^	2 (0)^ac^	2 (0)^bd^	<0.001
>39°C	1 (1)^a^	1 (0)^b^	1 (1)^c^	0 (0)^abc^	<0.001

* Viability measured according to the following the motility score: 0 paralyzed and dead, 1 very reduced motility, 2 reduced motility, 3 good motility. For each interaction, 15 adult stages of *F. hepatica* were evaluated in duplicate. The median and interquartile range in parenthesis of motility scores are presented;

** *p-value* calculated with the Kruskal-Wallis test and Dunn´s post-hoc test.

a,b,c,d Presence of equal letters symbolizes statistically significant difference.

### Cultivation

The Hedon-Fleig media maintained the viability of the adult stages of *F. hepatica* with a median life of 4 days, reaching maximum survival values of 6 days; while the RPMI and DMEM media kept *F. hepatica* alive with a median of 5 days, reaching maximum values of up to 7 days of life. All *F. hepatica* remained alive for at least 48 hours in all the culture media. Based on this result, all evaluations were performed for 48 hours. The evaluation of culture media during 48 hours of incubation showed that RPMI 1640 and DMEM media maintained the maximum viability (motility score 3) in contrast to Hedon-Fleig media (motility score 2; p<0.001). Regarding culture density, *F. hepatica* survived up to 48 hours incubation with high viability, with a minimum of 3 ml of culture media per adult fluke (*p* < 0.001), requiring media replacement for longer culture or to increase the initial volume of culture media. Also, no difference was found between 6- and 24-well culture plates (*p* > 0.05); *F. hepatica* could survive in both culture plates with high viability (motility score 3; Table 2).

**Table 2:** Assess of the *in vitro* viability of *Fasciola hepatica* according to the culture media, volume of the culture media, type of plate used and DMSO concentration.

**Variable**	**Condition**	**Motility score** ^*^ **at 48 hours of incubation**		***p-value*** ^**^
		**Median (iqr)** ^***^	**Min-max**	
**Culture media**	RPMI 1640^a^	3 (0)	3 - 3	<0.001
	DEMEN^b^	3 (0)	3 - 3	
	Hedon-Fleig^ab^	2 (0)	1 - 2	
				
**Volume of culture media used for a fluke**	2 ml^abc^	1 (0)	0 - 2	<0.001
	2.5 ml^d^	2 (1)	1 - 2	
	3 ml^ad^	3 (0)	3 - 3	
	4 ml^b^	3 (0)	3 - 3	
	5 ml^c^	3 (0)	3 - 3	
				
**Culture** ^†^ **plates**	1.9 cm^2^	3 (0)	3 - 3	0.999
	9.5 cm^2^	3 (0)	3 - 3	
				
**DMSO (% v/v)**	0^a^	3 (0)	3 - 3	<0.001
	0.2^b^	3 (0)	3 - 3	
	0.5^abc^	2 (0)	1 - 2	
	1^ab^	1 (1)	0 - 1	
	5^abc^	0 (0)	0 - 0	

* Viability measured according to the following the motility score: 0 paralyzed and dead, 1 very reduced motility, 2 reduced motility, 3 good motility. For each interaction, 5 adult stages of *F. hepatica* were evaluated in duplicate;

† 1.92cm^2^: Growth area per well corresponding to 24-well culture plates; 9.5cm^2^: Growth area per well corresponding to 6-well culture plates;

** *p-value* calculated with Kruskal-Wallis test and Dunn´s post-hoc test;

*** Median (interquartile range).

a,b,c,d Equal letters mean statistical difference.

The DMSO concentration added to the culture showed that *F. hepatica* remained viable (motility score 3) until 0.2% v/v at 48h of incubation, but higher concentration reduced the viability up to the total paralysis of flukes (*p* < 0.05; Table 2).

**Figure 1:**
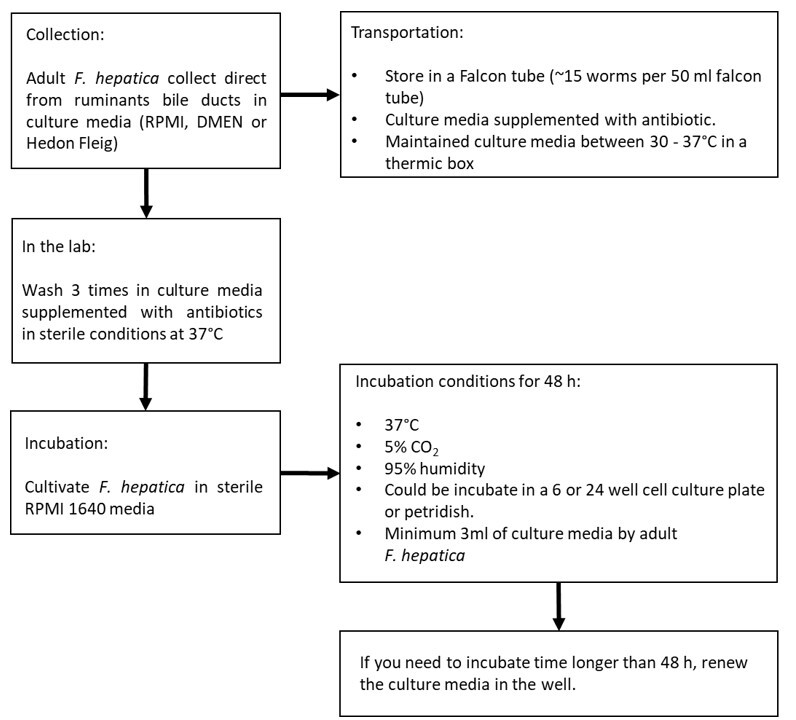
*In vitro* culture workflow of adult stage of *Fasciola hepatica*.

Based on these results, we propose a workflow for the cultivation of *F. hepatica* (Figure 1). It is recommended that the entire culture process be conducted under sterile conditions, as contamination could result in incubation failure. In addition, heat shock of the flukes must also be avoided during the entire transport, washing and incubation process.

## Discussion

According to our results, the transportation an incubation of *F. hepatica* can be carried out with various culture media. This diversity of media used is widely reported in the scientific literature (Singh et al., 2009; Duthaler et al., 2010; Tansatit et al., 2012). However, in this study, media such as RPMI 1640 and DMEM were found to be more suitable in maintaining *F. hepatica* with good viability under *in vitro* conditions, probably due to the diversity of its components (carbohydrates, amino acids, vitamins and minerals), necessary for parasite survival (Cantor, 2019).

Even though the DMEM and RPMI 1640 media showed great efficiency for transport and cultivation, the working group agreed that the RPMI 1640 media generated better motility of *F. hepatica* with respect to the DMEM media, both in transport and in incubation. However, they are very subtle differences, hard to establish on a motility scale that can even include some subjectivity. We also suggest washing the parasites with the culture media, to help maintain good viability of *F.hepatica* during this stressful process. Finally, the temperature is a fundamental factor for viability during the transport and washing process, since it is a simulation of *in vivo* conditions. Therefore, thermal shocks must be avoided, and it is suggested to keep the flukes at temperatures close to 37°C during the entire process.

*F. hepatica* was able to survive without difficulty in both the 6- and the 24-well culture plates. These results are based on the normal living conditions in the livers of naturally infected animals, where they live compressed in the bile ducts with other flukes, mineral deposits and the passage of the bile (Caravedo & Cabada, 2020). This condition was also documented in another study, where 12-well culture plates were successfully used for the incubation of *F. hepatica* (Duthaler et al., 2010). This evidence confirms the versatility of *F. hepatica* for *in vitro* cultivation in different types of culture plates.

DMSO is an organic solvent for polar and nonpolar solutions, widely used in pharmacology and toxicology studies (Modrzyński et al., 2019); nevertheless, DMSO can have toxic effects in blood and endothelial cells *in vitro* analysis at 0.6% concentration (Yi et al., 2017). We showed that DMSO begins to generate toxicity in *F. hepatica* at concentrations of 0.5% at 48 hours of incubation, thus, various *in vitro* studies in *Fasciola* spp. use a concentration of 0.1% of DMSO (Tansatit et al., 2012; Ullah et al., 2017), which we have shown to be a non-toxic concentration.

This study successfully identified optimal conditions for transport and culture of *F.hepatica*, but it has its limitations. Firstly, the flukes were acquired from a slaughterhouse, so we did not have access to information regarding treatment of the animals with antiparasitic drugs (albendazole, triclabendazole, clorsulon, etc.). Therefore, a motility score was applied in freshly collected flukes and before starting their incubation to ensure the selection of best viable flukes. In addition, flukes from multiple bovines were selected, to avoid sampling from the same possible treated animal. Secondly, the viability assessment could not be executed for more than 48 hours, due to the presence of some dead flukes. However, this evaluation period is enough to evaluate the behavior of different metabolites *in vitro* taking into account that time to peak drug concentration of many flukicidal drugs are 12 to 40 hours (Ortiz et al., 2014; Rehbein et al., 2024). Despite these challenges, we propose a protocol that maintains high viability of adult *F. hepatica* throughout the entire culture process. This protocol could be applied for the evaluation of new antiparasitic treatments *in vitro*.

## Conclusion

DMEM, RPMI 1640, Hedon-Fleig and PBS culture media kept *F. hepatica* viable during the transport process at approximately 37°C. In addition, the DMEM and RPMI 1640 media maintained *F. hepatica* with the best viability during incubation while the DMSO at 0.5% v/v reduced the viability of *F. hepatica* during the 48 hours of incubation.
